# Mutation C3256T of Mitochondrial Genome in White Blood Cells: Novel Genetic Marker of Atherosclerosis and Coronary Heart Disease

**DOI:** 10.1371/journal.pone.0046573

**Published:** 2012-10-02

**Authors:** Igor A. Sobenin, Margarita A. Sazonova, Maria M. Ivanova, Andrey V. Zhelankin, Veronika A. Myasoedova, Anton Y. Postnov, Serik D. Nurbaev, Yuri V. Bobryshev, Alexander N. Orekhov

**Affiliations:** 1 Laboratory of Medical Genetics, Russian Cardiology Research and Production Complex, Russian Ministry of Health and Social Care, Moscow, Russian Federation; 2 Laboratory of Cellular Mechanisms of Atherogenesis, Institute of General Pathology and Pathophysiology, Russian Academy of Medical Sciences, Moscow, Russian Federation; 3 Department of Clinical Investigations, Institute for Atherosclerosis Research, Skolkovo Innovative Centre, Moscow, Russian Federation; 4 Faculty of Medicine, University of New South Wales, Sydney, New South Wales, Australia; Medical University Hamburg, University Heart Center, Germany

## Abstract

This study was undertaken to examine the association between the level of heteroplasmy for the mutation C3256T in human white blood cells and the extent of carotid atherosclerosis, as well as the presence of coronary heart disease (CHD), the major clinical manifestation of atherosclerosis. Totally, 191 participants (84 men, 107 women) aged 65.0 years (SD 9.4) were recruited in the study; 45 (24%) of them had CHD. High-resolution B-mode ultrasonography of carotids was used to estimate the extent of carotid atherosclerosis by measuring of the carotid intima-media thickness (cIMT). DNA samples were obtained from whole venous blood, and then PCR and pyrosequencing were carried out. On the basis of pyrosequencing data, the levels of C3256T heteroplasmy in DNA samples were calculated. The presence of the mutant allele was detected in all study participants; the level of C3256T heteroplasmy in white blood cells ranged from 5% to 74%. The highly significant relationship between C3256T heteroplasmy level and predisposition to atherosclerosis was revealed. In individuals with low predisposition to atherosclerosis the mean level of C3256T heteroplasmy was 16.8%, as compared to 23.8% in moderately predisposed subjects, and further to 25.2% and 28.3% in significantly and highly predisposed subjects, respectively. The level of C3256T heteroplasmy of mitochondrial genome in human white blood cells is a biomarker of mitochondrial dysfunction and risk factor for atherosclerosis; therefore, it can be used as an informative marker of genetic susceptibility to atherosclerosis, coronary heart disease and myocardial infarction.

## Introduction

In a recent pilot study which was undertaken in order to see if any ultrastructural peculiarities in leukocytes in the blood of patients with atherosclerosis might exist, we noted a marked variability in structural appearance of mitochondria in leukocytes obtained from the blood of patients with atherosclerosis compared with that of healthy donors (unpublished data) (Figure S1). This observation prompted us to consider a possibility that the structural heterogeneity and structural alterations of mitochondria in leukocytes in the blood of patients with atherosclerosis might relate to mitochondrial mutations.

Numerous polymorphisms of the nuclear genome, which are believed to be genetic risk factors for atherosclerotic diseases, can help explaining for no more than 3% of the variability of clinical manifestations of atherosclerosis, such as coronary heart disease (CHD). At the same time, mutations of the mitochondrial genome remained out of focus for a long time, although they may play a pathogenic role in the formation of atherosclerotic lesions of human arteries causing various defects in the protein chains of some respiratory enzymes and transfer RNA (tRNA), synthesized directly in the mitochondria. This leads to a decrease in the concentration of these enzymes and their tRNA or total dysfunction, which contributes to the development of oxidative stress and increased the probability of occurrence and development of atherosclerosis [1].

Mitochondrial mutations can be both somatic and inherited through the maternal line. They are characterized by the phenomenon of heteroplasmy, which is defined as the presence of a mixture of more than one type of an organellar genome within a cell or individual. Mitochondrial DNA is present in hundreds to thousands of copies per cell and also has a very high mutation rate. New mtDNA mutations arise in cells, coexist with wild-type mtDNA, and segregate randomly during cell division. We have demonstrated recently that there are significant differences between unaffected intima of human aorta and atherosclerotic lipofibrous plaque in the level of heteroplasmy for point substitution C3256T, which is located in the nucleotide sequence of MT-TL1 gene coding for tRNA leucine [2].

Taking into account the pathogenic role played by human leukocytes in the formation of atherosclerotic lesions [3], we considered that it is reasonable to investigate the relationship between the mutation “load” of peripheral blood cells and susceptibility to atherosclerosis.

This study was undertaken to examine the association between the level of heteroplasmy for the mutation C3256T in human white blood cells and the extent of carotid atherosclerosis, and the presence of CHD as the major clinical manifestation of atherosclerosis.

## Results

Antropometric, clinical and biochemical data of 191 study participants are presented in Table 1. There were no significant differences in gender ratio as in healthy participants, as in CHD patients (P = 0.10). CHD patient were older than healthy participants; mean age was 70.0 (SD 8.7) and 63.5 (SD 9.0), respectively, P<0.001. The was no significant difference in fasting blood glucose levels, in spite of the higher proportion of Type 2 diabetic patients among CHD patients. The difference in SBP and DBP did not reach statistical significance, in spite of the higher proportion of hypertensive patients among CHD patients. The difference in lipid levels also did not reach statistical significance between non-CHD and CHD groups, this is partially due to statins taken by 25% CHD patients. None of antropometric or biochemical variables possessed an explanatory value for the difference in C3256T heteroplasmy, as was shown by regression and correlation analyses.

**Table 1:** Antropometric, clinical and biochemical data of study participants.

Variable	Total group, n = 191	Non-CHD controls, n = 146	CHD patients, n = 45	P for the difference
Age, years	65.0 (9.4)	63.5 (9.0)	70.0 (8.7)	<0.001 *
Gender, m:f	84∶107	60∶86	24∶21	NS (0.102)
BMI, kg/m^2^	26.9 (4.6)	26.6 (4.5)	27.7 (4.8)	NS (0.100) **
SBP, mm Hg	139 (17)	138 (17)	141 (18)	NS *
DBP, mm Hg	83 (11)	83 (10)	81 (13)	NS ***
Smokers, %	8.9	11.0	2.2	NS (0.057)
Hypertension, %	64.9	58.9	84.4	0.001
LVH, %	34.0	28.1	53.3	0.002
Diabetes, %	12.6	6.8	31.1	<0.001
Angina, %	24.1	0.0	100.0	<0.001
AMI in anamnesis, %	3.7	0.0	15.6	<0.001
Stroke in anamnesis, %	2.6	0.0	8.9	0.011
Family anamnesis for AMI, %	27.7	26.0	33.3	NS
Family anamnesis for HT, %	39.3	39.0	40.0	NS
Family anamnesis for T2DM, %	17.3	17.1	17.8	NS
Total cholesterol, mg/dl	239 (48)	240 (48)	235 (50)	NS *
Triglycerides, mg/dl	127 (60)	125 (60)	131 (60)	NS **
LDL cholesterol, mg/dl	148 (43)	148 (43)	145 (45)	NS *
HDL cholesterol, mg/dl	66 (15)	67 (15)	63 (16)	NS *
Fasting blood glucose, mmol/l	4.9 (1.2)	4.8 (1.2)	5.2 (1.6)	NS *
Statins, %	11.5	7.5	24.4	0.006
Plaque, score	0.83 (0.86)	0.73 (0.85)	1.16 (0.85)	0.003 **
Mean cIMT, µm	869 (167)	841 (150)	961 (189)	<0.001 *
Maximum cIMT, µm	1006 (213)	971 (185)	1117 (258)	<0.001 **
C3256T heteroplasmy, %	23.3 (14.7)	21.7 (13.4)	28.8 (17.3)	0.035 **

* - one-way ANOVA;

** - Mann-Whitney U-test;

*** - Welch test.

The mean level of C3256T heteroplasmy in white blood cells was 23.3% (SD 14.7), while its distribution was significantly different from normal (Kolmogorov-Smirnov test with Lilliefors's correction, P<0,001). The level of C3256T heteroplasmy ranged from 5% to 74%; therefore, the presence of the mutant allele was detected in all study participants. Median accounted for 18%, interquartile range was from 13% (2nd quartile) to 36% (3rd quartile).

There was statistically significant difference in C3256T heteroplasmy level between men and women; the mean values accounted for 20.8% (SD 15.1) and 25.4% (SD 14.1), respectively (P = 0.001).

**Figure 1:**
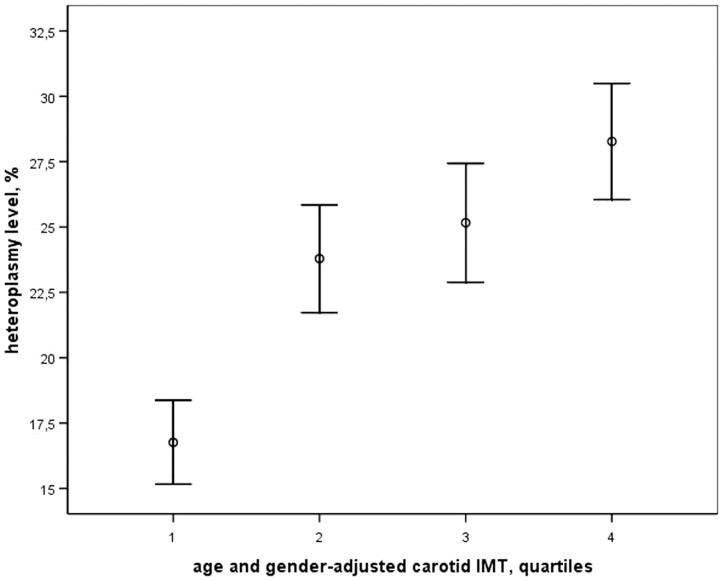
Graph showing the association of the level of C3256T heteroplasmy of mitochondrial DNA from white blood cells with cIMT quartiles. The data are presented as mean values and S.E.M.

The relationship between C3256T heteroplasmy and predisposition to atherosclerosis was evaluated. In individuals with low predisposition to atherosclerosis the mean level of C3256T heteroplasmy was 16.8% (SD 11.3), in moderate predisposition −23.8% (SD 14.3), in elevated predisposition −25.2% (SD 15.9), and in high predisposition −28.3% (SD 14.8) (Figure 1). One-way analysis of variance revealed a linear relationship between cIMT grade and the level of C3256T heteroplasmy (F = 5.674; P = 0.001); correlation coefficient between these parameters was 0.295 (P<0.001). The significant relationship between C3256T heteroplasmy level and cIMT grade was revealed also by Kruskal-Wallis analysis of variance (Chi-Square, 75.79, P = 0.004).

**Figure 2:**
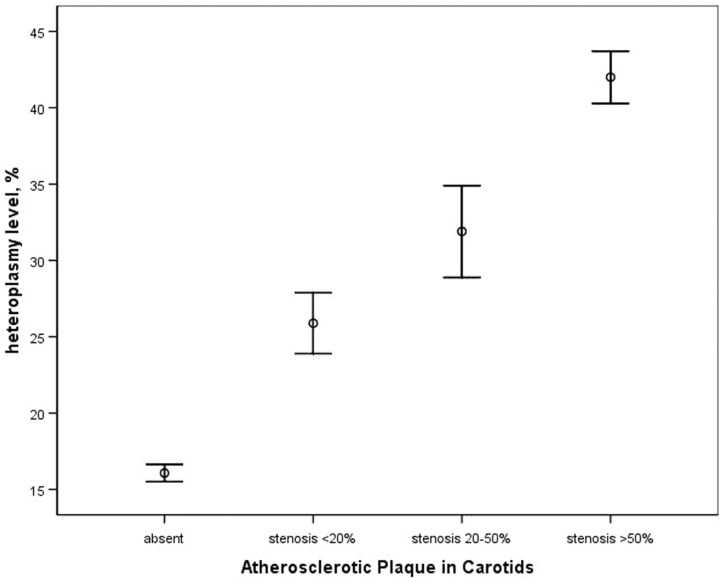
Graph showing the association of the level of C3256T heteroplasmy of mitochondrial DNA from white blood cells with the size of carotid atherosclerotic plaque. The data are presented as mean values and S.E.M.

Further the relationship between C3256T heteroplasmy and the severity of carotid atherosclerotic plaques was evaluated. These parameters were also related linearly (P<0.001), and correlation coefficient was 0.333 (P<0.001). The relationship between C3256T heteroplasmy level and presence of atherosclerotic plaques was also evaluated by Kruskal-Wallis H Test, which revealed significant association between these variables (Chi-Square, 17.20, P< 0.001). The data on the mean levels of the C3256T heteroplasmy related to the size of carotid atherosclerotic plaques are presented in Figure 2.

The relationship between C3256T heteroplasmy level and the presence of clinical manifestations of atherosclerosis was also assessed. The mean level of heteroplasmy in white blood cells from healthy persons was 21.7% (SD 13.4), while in CHD patients it accounted for 28.8% (SD 17.3), the difference was significant at P = 0.035. The relationship between C3256T heteroplasmy level and the presence of clinical manifestations was also evaluated by Kruskal-Wallis analysis of variance. The difference in mean levels of heteroplasmy between 3 groups (non-CHD study participants, CHD patients, and CHD patients – AMI survivors) was significant at P = 0.042 (Chi-Square, 6.33).

The small number of CHD patients had an acute myocardial infarction in anamnesis (n = 7); however, even for such small sample the difference C3256T heteroplasmy level was observed as compared to study participants who had no myocardial infarction history: 38.4% (SD 20.7) vs 22.8% (SD 14.1), respectively (P = 0.042). At the same time, in CHD patients who had no myocardial infarction history, the level of C3256T heteroplasmy accounted for 27.0% (SD 16.3), which looked like an intermediate value (one-way ANOVA: F = 6.142, P = 0.003; Kruskal-Wallis test: Chi-Square, 6.33, P = 0.042).

The ROC-curve analysis of sensitivity/specificity ratio was performed to evaluate diagnostic significance of C3256T heteroplasmy level, when high predisposition to atherosclerosis, or the presence of any atherosclerotic plaque in any visualized carotid segment, or the presence of CHD, or the history of myocardial infarction were taken as actual states. The results of analysis shown in Table 2 demonstrate that C3256T heteroplasmy level is of high diagnostic value for all actual states, except for the history of myocardial infarction, since due to the insufficient number of such patients the estimates of sensitivity and specificity of diagnostics were considered unreliable.

**Table 2:** Analysis of sensitivity/specificity ratio of C3256T heteroplasmy.

Actual state	Area under ROC-curve	Cut-off value of C3256T heteroplasmy level	Sensitivity	Specificity
High predisposition to atherosclerosis	0.625 (95%CI, 0.528–0.722, P = 0.012)	19.5%	63.6%	63.3%
The presence of any atherosclerotic plaque in any visualized carotid segment	0.675 (95%CI, 0.596–0.753, P<0.001)	17.5%	60.7%	63.1%
The presence of CHD	0.604 (95% CI, 0.500–0.707, P = 0.036)	19.5%	60.0%	62.3%
The history of myocardial infarction	0.726 (95%CI, 0.510–0.943, P = 0.042)	22.5%	71.4%	69%

**Figure 3:**
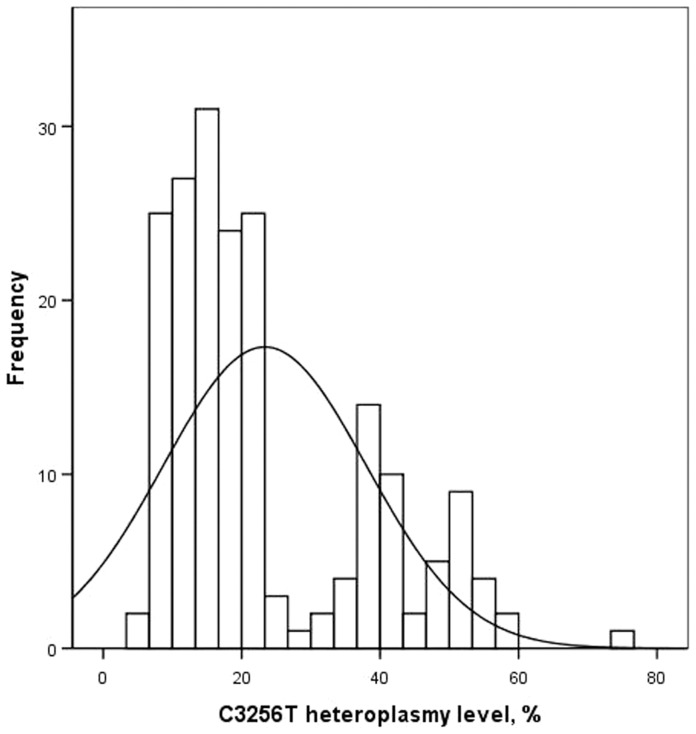
Histogram showing the distribution of the level of C3256T heteroplasmy of mitochondrial DNA from white blood cells. Theoretical normal distribution is shown by solid curve.

Since the distribution of the levels of C3256T heteroplasmy in the studied sample was different from normal, a two-step cluster analysis was performed (Figure 3). It turned out that there exist at least two different overlapping distributions of C3256T heteroplasmy in white blood cells, with the mean levels of 15.1% (SD 4.9; n = 138) and 44.9% (SD 8.2; n = 53). In the cross-tabulation analysis the belonging of study participant to the cluster 1 or 2 was used as a classification (nominal) variable. The results are presented in Table 3.

**Table 3:** Contingency of phenotypic and clinical manifestations of atherosclerosis with the level of C3256T heteroplasmy of mitochondrial genome.

Manifestation	Number of study participants		Chi-square	P-value	Statistical power (1-β)
	Low heteroplasmy level (cluster 1, n = 138)	High heteroplasmy level (cluster 2, n = 53)			
The presence of carotid atherosclerotic plaques	55 (39.8%)	52 (98.1%)	52.75	<0.001	100%
The presence of CHD	24 (17.4%)	21 (39.6%)	10.51	0.002	89%
The history of myocardial infarction	2 (1.4%)	5 (9.4%)	6.92	0.018	60%

It was found that in individuals with high levels of C3256T heteroplasmy (cluster 2), the relative risk of having atherosclerotic plaques in carotid arteries is 2.46 (95% confidence interval, 2.07–2.55), the relative risk of CHD −2.28 (95% confidence interval, 1.32–3.82), and the relative risk of myocardial infarction −6.51 (95% confidence interval, 1.16–47.98). The accuracy of diagnostics was 70.7%, 70.7% and 73.8%, respectively.

The level of C3256T heteroplasmy correlated with the age of study participants (r = 0.296; P<0.001), which demonstrates the possibility of accumulation of the mutant allele within an individual's life. This correlation was observed both in men (r = 0.312; P = 0.004), as in women (r = 0.259; P = 0.007).

Correlation of the level of C3256T heteroplasmy with traditional risk factors of CHD was revealed only in women for systolic blood pressure (r = 0.307; p = 0.001), and triglycerides (0.242; P = 0.012), but not in men.

## Discussion

The results of this study have showed that the mutation C3256T of mitochondrial DNA observed in human white blood cells is definitely associated with atherosclerosis at high level of statistical significance. It is noteworthy that such association is observed not only with the quantitative ultrasonographic characteristics of predisposition to atherosclerosis (intima-media thickness of common carotid arteries), but also with ultrasonographic signs of subclinical atherosclerosis (the presence of atherosclerotic plaques and their size), and with the clinical manifestations of atherosclerosis (coronary heart disease and myocardial infarction).

Thus, the level of C3256T heteroplasmy of mitochondrial genome in human white blood cells is a risk factor for atherosclerosis and can be used as an informative marker of genetic susceptibility to atherosclerosis, coronary heart disease and myocardial infarction. The relative risks, defined by this marker, exceed the diagnostic characteristics of the convenient phenotypic risk factors, which usually varies in the range of 1.4–1.8.

The analysis of the results of our study allowed us to determine the cut-off values of C3256T heteroplasmy with the risk of atherosclerosis and its clinical manifestations. All tested approaches to assessment of sensitivity and specificity, using high susceptibility to atherosclerosis, the presence of atherosclerotic plaques, the presence CHD or the history of myocardial infarction as actual states, provided similar estimates, which averaged for ∼20%.

The point nucleotide substitute C3256T is described as a mutation of the mitochondrial genome associated with the development of MELAS-syndrome, a neurodegenerative mitochondrial disease. In this disease, mitochondrial myopathy, encephalopathy, lactic acidosis, as well as stroke-like states may be observed [4], [5]. This mutation was also found to be contingent with Type 2 diabetes mellitus of mitochondrial origin [6].

Nevertheless, the association of C3256T mutation with atherosclerosis has not yet been demonstrated. This may be due to some methodological problems: the vast majority of the nucleotide sequence analysis methods do not allow for precise quantitative measurement, and indicate only the presence of the mutant allele or provide semi-quantitative assessment of the proportion of mutant alleles in biological samples [7]–[9]. At the same time, homoplasmy is not known for C3256T mutation; that is, the mutant allele should be present in all mtDNA samples, which is typical for heteroplasmy and complicates analysis of the relationship of this mutation with any human disease. The results of our study also demonstrated that C3256T heteroplasmy was revealed in 100% participants.

The other method of diagnostics, based on quantifying of the encoded product and/or metabolites also has significant limitations, since it only suggests the higher or lower expression of the mutant allele [10].

The method of quantification of the proportion of mutant allele (the level of heteroplasmy) developed by us earlier has sufficient accuracy and reproducibility, since it is based on pyrosequencing of amplified DNA fragments containing the polymorphic site. The calculation of the share of the mutant allele is made by comparing the peaks of fluorescence for nucleotides, which are defined in a given position of oligonucleotide sequence [2]. The use of this methodological approach allowed us to estimate the variability of C3256T heteroplasmy in the studied sample, as well as to study the association of this genetic marker with a predisposition to atherosclerosis, being described also by quantitative methods.

Mutation C3256T is located in MT-TL1 gene (codon recognizing UUR) of mitochondrial genome, and is the coding sequence of tRNA leucine. The mutation is realized at the cellular level as a reduced amount of cellular organelles and the impaired protein synthesis [11]–[13]. Obviously, clinical and phenotypic manifestations of the mutation depend on the level of heteroplasmy.

High prevalence of C3256T mutation in population allows suggesting that it is maternally inherited. On the other hand, we have revealed a significant correlation between the level of C3256T heteroplasmy and age, which supports also the somatic nature of mutation: in any case, there is an increase in the proportion of mutant alleles of the mitochondrial genome of human white blood cells with age. It is not known whether the processes of accumulation of the mutant allele occur in other tissues of the human body. Preferential survival of somatic cells or progenitor cells with higher content of the mutant allele in the mitochondrial DNA is also possible, although this assumption contradicts the fact of association of C3256T mutation with neurodegenerative diseases and atherosclerosis, which reduce longevity.

By present, nothing is definitely known about the exact mechanisms of atherogenic action of C3256T mutation of mitochondrial genome, as well its role in silent myocardial impairment. It is obviously necessary to investigate the effects of this mutation on functional parameters like mitochondrial oxygen consumption, ROS production, levels of antioxidants, membrane potential, mitochondrial mass, etc. This should also include the studies with a special emphasis on specific atherosclerosis-related parameters, such as intracellular LDL traffic, intracellular cholesterol accumulation, synthesis and secretion of proinflammatory cytokines and chemokines, expression of genes responsible for inflammation and apoptosis.

Blood-derived cells, especially monocyte-macrophages, play an important role in atherogenesis [3], [14]–[17]. While migrating to the subendothelial space of elastic and muscular-elastic arteries, they are actively involved in the processes of inflammation and the formation of atherosclerotic lesions [3], [14]–[17]. We can assume that the higher the level of heteroplasmy of mitochondrial DNA, the higher the probability of inhibiting the functional activity of monocytes. If the mutation occurs in the coding region of gene, as in the case of C3256T mutation, it can lead to local pathological reactions, thus accelerating the development of atherosclerosis.

The results of the present study suggest that the C3256T heteroplasmy of mitochondrial DNA from white blood cells is a biomarker of genetic predisposition to the development of atherosclerosis and related clinical manifestations. It can be speculated that C3256T heteroplasmy is also a biomarker of mitochondrial dysfunction, although additional studies should be performed to support this hypothesis. It should be noted that this study has some limitations. First, even if the sample size was sufficient to detect significant differences in the level of C3256T heteroplasmy between CHD-free individuals and CHD patients (the statistical power was 81.2% at α<0.05), but it was rather small to avoid type 2 errors in the analysis of differences between persons with or without history of myocardial infarction (the statistical power was only 63.0% at α<0.05). It should also be noted that the sample was taken from ethnically heterogeneous population of Moscow inhabitants of senior and elderly ages. Therefore, at present there is insufficient evidence to interpolate the results of this study to other populations and age groups. Finally, the given study was cross-sectional, and the assessment of actual risk of atherosclerosis and cardiovascular disease due to the presence of a high level of C3256T heteroplasmy requires further prospective studies.

## Materials and Methods

This study was kept in accordance with the Helsinki Declaration of 1975 as revised in 1983. It was approved by the ethics committee of the Institute of Atherosclerosis Research, Moscow, Russia. In total, 191 participants were recruited in the study (84 men, 107 women) aged 65.0 years (SD 9.4); among them 45 participants (24%) had coronary heart disease (CHD). All participants gave their written informed consent prior to their inclusion in the study.

High-resolution B-mode ultrasonography was used for carotid arteries imaging to assess the extent of carotid atherosclerosis. The protocol of ultrasound examination involved the scanning of the right and left common carotid artery and the area of the carotid sinus (bulb) as high up as possible [18]. Three fixed angles of interrogation were used (anterolateral, lateral, and posterolateral). Images were focused on the posterior wall of the artery. The B-mode ultrasound system (SSI-1000, SonoScape, China) used a 7.5 MHz linear array probe. The measurements were always performed at 10-mm section of common carotid artery adjacent to the carotid bulb. The carotid intima-media thickness (cIMT) of the posterior wall was measured as the distance from the leading edge of the first echogenic (bright) line to the leading edge of the second echogenic line. The measurements were carried out with M′Ath computer software (IMT, France). The mean of three measurements (in anterolateral, lateral, and posterolateral positions) was considered to be the integral IMT estimate. Reproducibility of cIMT measurements was assessed according to the protocol of IMPROVE Study [19].

The degree of susceptibility to atherosclerosis was estimated by using interquartile cIMT values derived previously from separate Moscow population sample of 1287 participants (429 men, 858 women) free of CHD (Table 4); none of them belonged to the described set of 191 participants of the present study. The analysis of cIMT variation had shown that both samples were taken from the same parent population, in spite of the fact that 45 of 191 had CHD, and cIMT values were significantly higher in those CHD patients. This approach allowed distinguishing persons predisposed or not predisposed to atherosclerosis (Table 3). We considered persons belonging to the lowest quartile of age-adjusted cIMT distribution as non-predisposed to atherosclerosis, and those belonging to the highest quartile as highly predisposed to atherosclerosis. The belonging to the 2nd or the 3rd quartiles of cIMT distribution was regarded as a moderate or elevated susceptibility, respectively.

**Table 4:** The age-related borderlines for distribution of mean cIMT in Moscow population.

	Age, years			
	<50	51–60	61–70	>70
Men				
2nd quartile, µm	660	740	830	840
3rd quartile, µm	745	810	910	930
4th quartile, µm	800	910	990	1060
Women				
2nd quartile, µm	605	665	760	825
3rd quartile, µm	665	735	830	895
4th quartile, µm	740	815	920	990

Additionally, the presence and the size of atherosclerotic plaques in any visualized segment of carotid arteries was evaluated by 4-point scale (0 - no atherosclerotic lesions were observed; 1–2 - elevated atherosclerotic plaques taking up to 20% or 20 to 50% of lumen diameter, respectively: 3 - hemodynamically significant atherosclerotic plaques taking more than 50% of lumen diameter).

DNA samples were obtained from whole venous blood using commercially available kits for DNA extraction (BioRad, England). For the amplification of fragments of mitochondrial DNA by polymerase chain reaction (PCR) method followed by pyrosequencing, the corresponding primers and conditions were used, as described elsewhere [2]. The nucleotide sequences for biotinylated forward primer, reverse primer, and sequence primer were AGGACAAGAGAAATAAGGCC, ACGTTGGGGCCTTTGCGTAG, and AAGAAGAGGAATTGA, respectively. The buffer solution for PCR contained 2.5 mM MgCl_2_; annealing and extension phases of PCR were held at 55°C and 72°C, respectively. To quantitatively evaluate mutant allele, a method of pyrosequencing was adapted for conditions where both normal and mutant alleles were present in a biological specimen [2], [20]–[22]. Briefly, the defective allele was quantified by analyzing the peak heights in the pyrogram of one-chained PCR-fragments of a mitochondrial genome. The levels of C3256T heteroplasmy in DNA samples were calculated, taking into account the expected sequence and the dimension of peaks for the homozygotes possessing either 100% of the normal or 100% of the mutant allele, as described elsewhere [2].

Statistical analysis was performed using the SPSS v. 14.0 software package (SPSS Inc., USA). The methods of analysis of variance, cross-tabulation analysis, and correlation analysis by Spearman and Pearson were used. Search for factors, that had a significant influence on cIMT or C3256T heteroplasmy, was based on correlation coefficients. If both variables were interval or dichotomous, Pearson's correlation coefficient was used. If at least one variable was categorical, Spearman correlation coefficient was used. The comparisons of mean values for continuous variables were performed using one-way ANOVA, or U-test by Mann-Whitney, or Welch's test; the choice depended of normality of distribution and equality of dispersions; comparisons for categorical variables were made by chi-square Pearson's test [23]. Due to the non-normality of C3256T heteroplasmy distribution, non-parametric Kruskal-Wallis H Test was performed to estimate differences between groups [23]. Estimation of statistical power has been carried out using G*Power ver. 3.1.3 software package [24]. Post hoc procedure, aimed at computing achieved statistical power with given α, sample size and effect size, was performed. The data are presented in terms of mean and SD. The significance of differences was defined at the 0.05 level of confidence.

## Supporting Information

Figure S1 **Different ultrastructural appearances of mitochondria in leukocytes obtained from patients with atherosclerosis (A-D).** (**A**): A mitochondrion with well-defined cristae and well-preserved surrounding membranes (“intact” appearance). (**B**, **C**): Mitochondria with reduced numbers of cristae and the oedema of the mitochondrial matrix. (**D**): A mitochondrion displaying signs of damage; Note the oedema of the mitochondrial matrix and the presence of a myelin-like structure in the mitochondrial matrix (**A**-**D**): Electron microscopy. Scales  = 200 nm.
(TIF)
