# Supplementary figures and images for: Mutation C3256T of Mitochondrial Genome in White Blood Cells: Novel Genetic Marker of Atherosclerosis and Coronary Heart Disease
Source: PLoS One. 2012 Oct 2;7(10):e46573. doi: 10.1371/journal.pone.0046573 (PMC3462756; doi:10.1371/journal.pone.0046573)

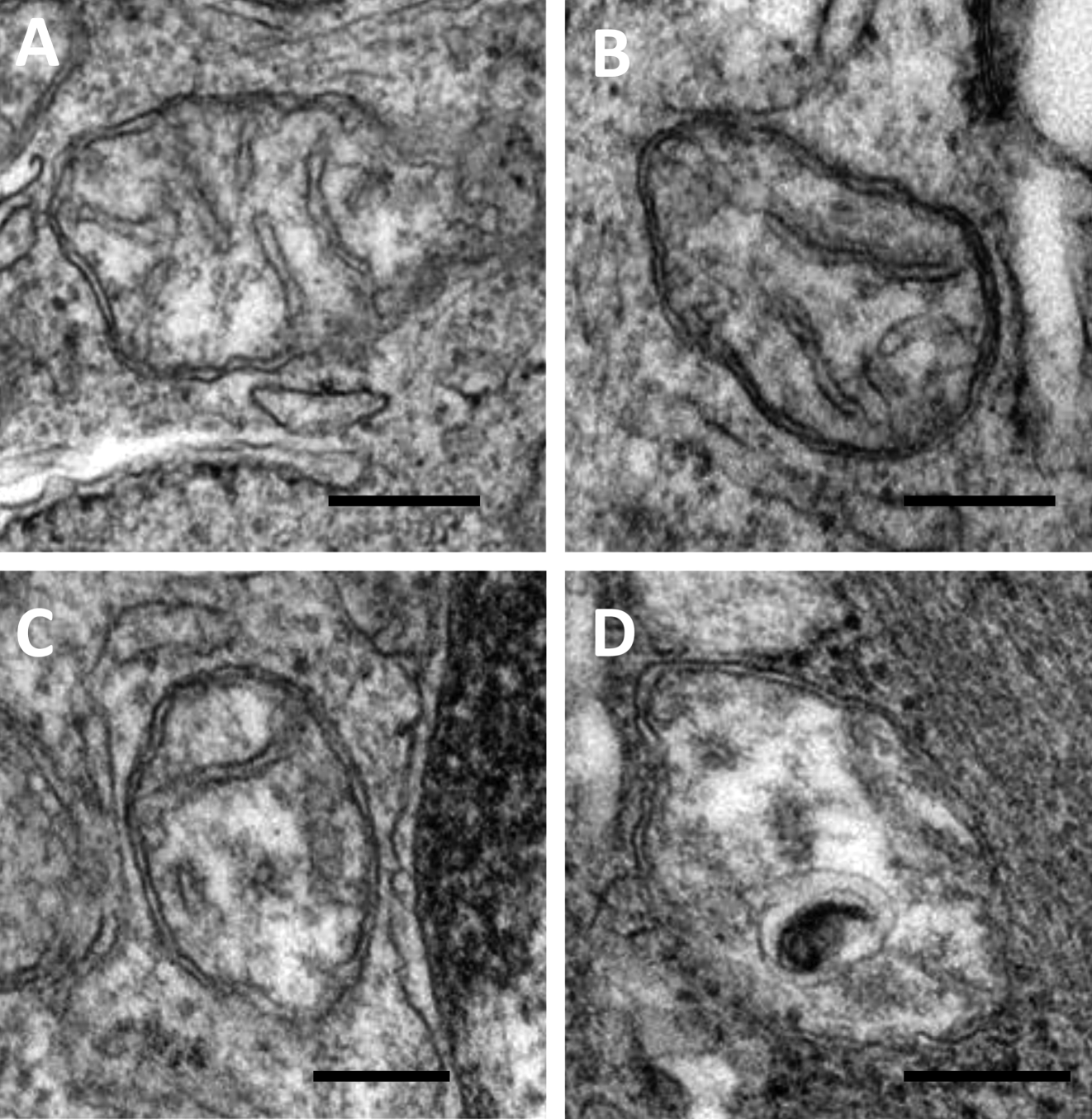

Supplement: Figure S1 — Different ultrastructural appearances of mitochondria in leukocytes obtained from patients with atherosclerosis (A-D). (A): A mitochondrion with well-defined cristae and well-preserved surrounding membranes (“intact” appearance). (B, C): Mitochondria with reduced numbers of cristae and the oedema of the mitochondrial matrix. (D): A mitochondrion displaying signs of damage; Note the oedema of the mitochondrial matrix and the presence of a myelin-like structure in the mitochondrial matrix (A-D): Electron microscopy. Scales = 200 nm. (TIF) [file pone.0046573.s001.tif]
